# Editorial: Emerging fungal pathogens: perspectives

**DOI:** 10.3389/ffunb.2024.1369062

**Published:** 2024-02-14

**Authors:** Danielly Corrêa-Moreira, Barbara de Oliveira Baptista, Domenico Giosa, Manoel Marques Evangelista Oliveira

**Affiliations:** ^1^ Laboratory of Taxonomy, Biochemistry and Bioprospecting of Fungi, Oswaldo Cruz Institute, Oswaldo Cruz Foundation, Rio de Janeiro, Brazil; ^2^ Laboratory of Malaria Research, Oswaldo Cruz Institute, Oswaldo Cruz Foundation, Rio de Janeiro, Brazil; ^3^ Department of Chemical, Biological, Pharmaceutical and Environmental Sciences, University of Messina, Messina, Italy

**Keywords:** immune response, emerging fungal pathogens, climatic changes, One Health, WHO critical group

Fungi constitute an essential and diverse component of most of the Earth’s ecosystems. They are eukaryotic organisms morphologically classified into yeast-like and filamentous forms, and both forms have high and varied functional diversity, as well as great variation in dispersion modes. It is estimated that the number of fungal species range from 11.7 to 13.2 million based on high-throughput sequencing. Nevertheless, only 150,000 fungal species have been described thus far, establishing different types of relationships, such as symbiotic, commensal, latent, or pathogenic (Bahram and Netherway, 2022; Hyde, 2022).

Mycoses are conditions in which fungi overcome hosts’ resistance barriers and cause infections. Billions of the world’s population are believed to be infected with pathogenic fungi, of which 1.7 million deaths are recorded each year, and we consider this number to be underreported despite being almost nine times higher than the number of deaths from bacterial tuberculosis, for example. However, until 2014, Kohler et al. (2014) believed that “few among the millions of fungal species fulfill four basic conditions necessary to infect humans: high temperature tolerance, ability to invade the human host, lysis and absorption of human tissue, and resistance to the human immune system”.

Mycoses are considered neglected diseases since their burden is most significant in tropical countries with an increase in the number of pathogenic species, and consequently, more underfunded than bacterial or viral diseases (Pathakumari et al., 2020; Burgess et al., 2022; Ahmed et al., 2023). Diverse clinical manifestations have been reported, ranging from self-limiting acute pulmonary syndrome and skin lesions in immunocompetent individuals to inflammatory diseases and life-threatening severe infections in immunosuppressed patients. It is essential to mention that the outcome of infection depends on the balance of the relationship of the host (immune response, site of infection) and pathogen (virulence factors, drug resistance).

Immunosuppression is an important factor in defining the outcome of fungal infection. The increase in the number of susceptible individuals, mainly due to AIDS, chemotherapy and organ transplantation, diabetes and autoimmune diseases, and treatment with broad-spectrum antibiotics or invasive medical procedures, as well as a recently identified group of patients with COVID-19 who received corticosteroids/immunosuppressant drugs, contributed to the significant increase in the global incidence of invasive fungal infections over the last 50 years (Hoenigl et al., 2022; Loh and Lam, 2023).

All these immunosuppressant conditions have highlighted the importance of fungal infections over time. However, the number of these diseases in immunocompetent patients has increased in the last decades. One of the critical issues to be addressed is the emergence of opportunistic fungi that have acquired the capacity to cause infections in these people, mainly due to their adaptation to climate changes, globalization, urbanization, habitat disturbance, loss of biodiversity, and resistance due to excessive use of antifungal agents. Many species, restricted to the “environmental niche” and considered to have no or low virulence, have been described as causal agents of various fungal infections, including causing deaths (Spallone and Schwartz, 2021).

In this scenario, there is an urgent need to develop new strategies to reduce, treat, or prevent fungal infections. An effective vaccine would be a powerful weapon, but no licensed vaccine is available to date. The main challenges in developing a fungal vaccine concern the molecular complexity of fungal pathogens and their immunological targets, the ability to evade host immune responses, and the immunological tolerance to commensal species, as well as induce a safe and efficacy immune response in immunocompromised or immunosuppressed individuals. Despite these obstacles, several vaccine candidates are in preclinical trials, and three are in human clinical trials, showing that a fungal vaccine is not an impossible goal. However, more attention from investors and pharmaceutical industries, research funding, and the use of new technologies are needed for an approved fungal vaccine for humans in the future (Pathakumari et al., 2020; Loh and Lam, 2023). Thus, understanding not only the mechanisms of the immune response to fungal infections but also the biological behavior of these organisms in response to all these anthropogenic actions is critical to the management of their prevention and control.

In 2022, the World Health Organization (WHO) released the first-ever list of health-threatening fungi (fungal priority pathogens list, or the FPPL), containing 19 groups of human fungal pathogens that are associated with a severe risk of mortality or morbidity (Loh and Lam, 2023). This list includes reemerging pathogens classified in the critical priority group as top-ranking. One example is *Cryptococcus neoformans*, a causal agent of cryptococcal meningitis that affects 194,000 people, with 147,000 deaths (Denning, 2024). Cryptococcosis is the second leading cause of death in people living with HIV (Zhao et al., 2023), but the fungus is also described as capable of causing endocarditis in immunocompetent people (McGuire and Walter, 2022). *Candida albicans* is well described as a species that has intrinsic resistance to several antifungals and can cause candidemia that affects 1,565,000 people annually, causing 995,000 deaths, due to its ability to form biofilm on catheters and prosthetic devices (Pathakumari et al., 2020; Denning, 2024). *Aspergillus fumigatus* is another species that has reemerged as a causal agent of pulmonary aspergillosis associated with COVID-19, influenza, and chronic obstructive pulmonary disease (COPD), with an incidence of 1,837,272 cases and 340,000 deaths (Spallone and Schwartz, 2021; Fisher and Denning, 2023; Denning, 2024).

These formers are described as reemerging because they have been recognized by clinicians long ago and may be increasing in prevalence due to a change in the susceptible population. However, despite the wide range of fungal pathogens that deserve attention in the context of global public health, it is necessary to highlight fungal infections caused especially by *Candida auris*, a new drug-resistant human pathogen, first isolated in 2009 and possibly the first pathogenic fungus emerging from human-induced global warming (Casadevall et al., 2019).

*C. auris* emerged simultaneously on three continents, with clades genetically distinct (clade I: South Asia, clade II: East Asia, clade III: Africa, and clade IV: South America), but it has a wide distribution in Latin America. In addition to clade IV, clade I in Brazil and Chile (Moreno et al., 2019; de Almeida Jr. et al., 2021) and clade III in Argentina (Garcia-Effron, 2023) have been described previously. Furthermore, the widespread use of antifungals brings to light the problem of the intrinsic resistance of these pathogens to different classes of antifungals, such as fluconazole, whose resistance is reported in more than 90% of isolates from clades I and III and about half of clade IV isolates. To a lesser extent, resistance against polyenes and echinocandins has also been demonstrated. On the other hand, isolates from clade II are susceptible to azole derivatives and other common antifungals, and in Brazil, where isolates from clades I and IV circulate, no resistance to any of these classes of antifungals has been observed (de Melo et al.; de Almeida Jr. et al., 2021).

As previously mentioned, mycoses are neglected diseases and are often underreported due to the scientific medical profession’s lack of knowledge of their causal agents. In this context, despite the publication of the WHO FPPL, classically opportunistic fungi have been reported as agents of infections in immunocompetent patients. Recently, articles were published describing *Fusarium solani* as the causal agent of an outbreak of meningitis in Mexico and USA, both in immunosuppressed and immunocompetent patients (Hoenigl et al., 2023; García-Rodríguez et al., 2024). In 2023, our group also published a case report of meningoencephalitis caused by *Penicillium chrysogenum*, a fungus widely distributed in nature, which, like *C. auris*, present in the high-priority group, acquired the ability to adapt to temperatures and cause diseases in healthy hosts (de Oliveira et al., 2023).

Some factors may contribute to the emergence and reemergence of these fungal pathogens, the main of which are human actions that result in climate change and induce fungal adaptative thermotolerance by reducing the difference between environmental and mammalian temperatures (Casadevall et al., 2019). In addition, the ecological imbalance evidenced by the SARS-Cov-2 pandemic modified the global panorama. Thus, with this background, this editorial reinforces the importance of improving One Health strategies that consider humans, animals, and the environment not as a fragmented system but as part of a whole, in which all actions, behaviors, and organisms are interconnected, and in which everyone bears, directly or indirectly, the consequences of decisions made.

## Author contributions

DC: Conceptualization, Writing – original draft, Writing – review & editing. BB: Writing – review & editing. DG: Writing – review & editing. MO: Conceptualization, Funding acquisition, Project administration, Resources, Writing – review & editing.
